# Latent toxoplasmosis impairs learning and memory yet strengthens short-term and long-term hippocampal synaptic plasticity at perforant pathway-dentate gyrus, and Schaffer collatterals-CA1 synapses

**DOI:** 10.1038/s41598-023-35971-2

**Published:** 2023-06-02

**Authors:** Samira Choopani, Bahereh Kiani, Shayan Aliakbari, Jalal Babaie, Majid Golkar, Hamid Gholami Pourbadie, Mohammad Sayyah

**Affiliations:** 1 Department of Physiology and Pharmacology, Pasteur Institute of Iran, Tehran, Iran; 2 Department of Biology, Damghan University, Damghan, Iran; 3 Department of Parasitology, Pasteur Institute of Iran, Tehran, Iran

**Keywords:** Parasitology, Mechanisms of disease

## Abstract

Investigating long-term potentiation (LTP) in disease models provides essential mechanistic insight into synaptic dysfunction and relevant behavioral changes in many neuropsychiatric and neurological diseases. *Toxoplasma (T) gondii* is an intracellular parasite causing bizarre changes in host’s mind including losing inherent fear of life-threatening situations. We examined hippocampal-dependent behavior as well as in vivo short- and long-term synaptic plasticity (STP and LTP) in rats with latent toxoplasmosis. Rats were infected by *T. gondii* cysts*.* Existence of REP-529 genomic sequence of the parasite in the brain was detected by RT-qPCR. Four and eight weeks after infection, spatial, and inhibitory memories of rats were assessed by Morris water maze and shuttle box tests, respectively. Eight weeks after infection, STP was assessed in dentate gyrus (DG) and CA1 by double pulse stimulation of perforant pathway and Shaffer collaterals, respectively. High frequency stimulation (HFS) was applied to induce LTP in entorhinal cortex-DG (400 Hz), and CA3-CA1 (200 Hz) synapses. *T. gondii* infection retarded spatial learning and memory performance at eight weeks post-infection period, whereas inhibitory memory was not changed. Unlike uninfected rats that normally showed paired-pulse depression, the infected rats developed paired-pulse facilitation, indicating an inhibitory synaptic network disruption. *T. gondii*-infected rats displayed strengthened LTP of both CA1-pyramidal and DG-granule cell population spikes. These data indicate that *T. gondii* disrupts inhibition/excitation balance and causes bizarre changes to the post-synaptic neuronal excitability, which may ultimately contribute to the abnormal behavior of the infected host.

## Introduction

Toxoplasmosis, is a parasitic disease caused by the obligate intracellular protozoan Toxoplasma gondii (*T. gondii*). The latest epidemiological reports count *T. gondii* infection as one of the most prevalent infectious disease in humans with an average global seroprevalence rate of 25.7%^1^.

Tachyzoites are the rapidly-multiplying form of *T. gondii*, with capability to invade the excitable cells in skeletal muscles and nerves in which they differentiate to encysted bradyzoites. Bradyzoites are the semidormant, slowly-dividing form of the parasite, which persist inside the cells for the entire life of the host and form the chronic latent phase of toxoplasmosis.

*T. gondii *exerts extraordinary changes in mentality and behavior of the infected host. Human studies have reported association between toxoplasma infection and dangerous behaviors such as suicide intents^2–4^, and driving accidents^5,6^. In mice, toxoplasma infection is associated with loss of inherent fear of predators^7^. Alterations in motor and learning performance, memory, affability, ascendancy, mate choice, exploratory behaviors and anxiety are also derived by *T. gondii* in animals^8^. In line with animal findings, the link between *T. gondii* seropositivity and anxiety disorders^9^, schizophrenia^6^, and dementia^10^ is reported in humans. Although these behavioral changes have been considered side effects of neuroinflammation caused by the parasite^7,11,12^, targeted modification of signaling at synapse and neurotransmitter levels are also a possible mechanism, supported by thorough studies^13–15^. In line with this, alterations in both glutamatergic and GABAergic neurotransmission are found in brain of mice with chronic *T. gondii* infection^13,16^. Furthermore, a significant decline in number of key synaptic markers for pre- and post-synaptic function, synaptophysin and postsynaptic density protein 95 (PSD-95/Dlg4)^17^, glutamatergic synapse components EAAT2, Shank3, and the receptor subunits GluA1, GluA2, GluN1, GluN2B^14^ are reported in the hippocampus of mice with chronic *T. gondii* infection.

Experience-dependent alterations in the strength of synaptic transmission between neurons play an essential role in survival of animal, and enables it to manage surrounding threats, and to forage. Thus, synaptic connections in the brain are dynamically remodeled in response to externally-triggered neuronal activity, and incorporate momentary experiences into perpetual memory traces^18^. This process is called synaptic plasticity: the activity-dependent changes in the strength or efficacy of synaptic transmission^19^. Synaptic plasticity is disrupted in variety of psychiatric disorders such as anxiety^20^, cognitive disabilities^21^, and mental health (perception, cognition, motivation, and the regulation of emotion) disorders^22^.

The hippocampal formation, consisting of the cornu ammonis (CA) 1, CA2, CA3, dentate gyrus (DG), and subiculum, has been implicated in cognitive processes^23^, anxiety function and integrating information from heterogeneous conceptual, contextual and topographical sources with sensory modalities^24^.

To the best of our knowledge, no electrophysiological study has assessed synaptic transmission and plasticity in the *T. gondii*-infected rodents. Recording extracellular potentials in vivo is a method that offers unique separation of spatial location of brain signals in real time. This approach advances our understanding of how synaptic plasticity mediates both adaptability and pathological experience-dependent flexibility in diseases which are accompanied by behavioral and mental alterations, such as toxoplasmosis.

In this study, we examined status of STP and LTP in rats with chronic toxoplasma infection, using evoked-field potential recording in vivo at perforant pathway-DG, and Schaffer collaterals-CA1 synapses of hippocampal trisynaptic circuit.

## Materials and methods

### Animals

Male Wistar rats (90–100 g, 4 weeks old, Pasteur Institute of Iran, n = 62) were housed in groups of 4 rats in standard polypropylene cages in a room with controlled temperature (23 ± 2.0 °C) and 12 h light/dark cycle (6:00–18:00). They were fed ad libitum with rodent’s chow and free access to drinking water. Animals were randomly divided to different experimental groups. All experiments were conducted during light phase.

Study was performed according to guidelines of Institutional Animals Ethics Committee of Pasteur Institute of Iran and Council Directive 2010/63EU of the European Parliament, and the Council of 22 September 2010 on the protection of animals used for scientific purposes. The authors complied with the ARRIVE guidelines. All experimental protocols were approved by Institutional Animals Ethics Committee of Pasteur Institute of Iran (License no. IR.PII.REC.1396.24).

### Infecting rats with *T. gondii*

Rats were infected with a type II strain of *T. gondii*, according to the previously described method^25^. Animals were inoculated by intraperitoneal (i.p.) injection of 500 cysts in 0.2 ml of phosphate buffer solution (PBS). A sham group was considered in the experimental groups, in which rats received PBS instead of cysts. At 4 and 8 weeks after inoculation, whole brain of rats were harvested for DNA extraction and parasite load determination.

### Molecular diagnosis of toxoplasmosis

REP-529 genomic sequence with 200–300 repetitions is one of the repetitive sequences of toxoplasma genome. Therefore, it is a suitable target for the diagnosis of toxoplasma infection^26^. The set of primers and probe was designed using AlleleID software version 5/7 (RRID: SCR_014790) and according to the published sequence of REP-529 sequence in NCBI (NCBI Nucleotide, RRID: LN714508). The RT-qPCR method was used to detect the copy number of REP-529 in the rat brain according to the previous method^27^. Briefly, the PCR product of REP-529 fragment was cloned in pTZ57R/T plasmid. The number of plasmids present in each microliter of the solution was calculated. Plasmid pTZ57R-REP529 was used to prepare standard 10-fold dilutions (10^1^, 10^2^, 10^3^ and 10^4^ plasmid/μl) in triplicates to draw the standard curve. A standard curve was used to obtain Cts and the number of copies of the REP-529 genomic sequence in the brain. Negative and positive controls were included in each test. The negative control was the PCR reaction without template DNA. As a positive control, genomic DNA purified from tissue cysts was used.

### Spatial and avoidance learning and memory tasks

Morris water maze was used to assess spatial learning and memory of animals^28^. In brief, in order to assess animals learning, they were released into a water tank to find a fixed hidden submerged platform in 60 s. They went through training sessions of 4 trials for 3 consecutive days. For evaluation of memory, 24 h after the last training session, the animals went through a probe test in which no platform was placed under the water, and the animals were expected to find the target quadrant. In the last stage, to test the visual ability of the participating rats, they were released into the water to find a visible elevated platform which was covered with a bright aluminum foil. The parameters of escape latency, traveled distance, time spent in target quadrant, and swim velocity were recorded for subsequent analysis.

The avoidance learning and memory was assessed by shuttle-box apparatus according to the method that is published before^29^. After habituating the animal with chamber, it was placed in the bright chamber with the guillotine door open. Once the animal entered the dark chamber, an electrical shock (1.5 mA, 50 Hz, for 1.5 s) was applied to the soles of feet via rod floor of the chamber. The animal was removed after 20 s. After 2 min, rat was again placed in the bright chamber with the guillotine door open. If the animal did not enter the dark room in 2 min, the avoidance memory is consolidated. The next day, in order to assess the memory recall, the animal was placed in the bright chamber, and was monitored for 5 min. The initial latency, step-through latency and the time spent in the dark chamber were recorded.

### Evoked field potential recording

**Figure 1:**
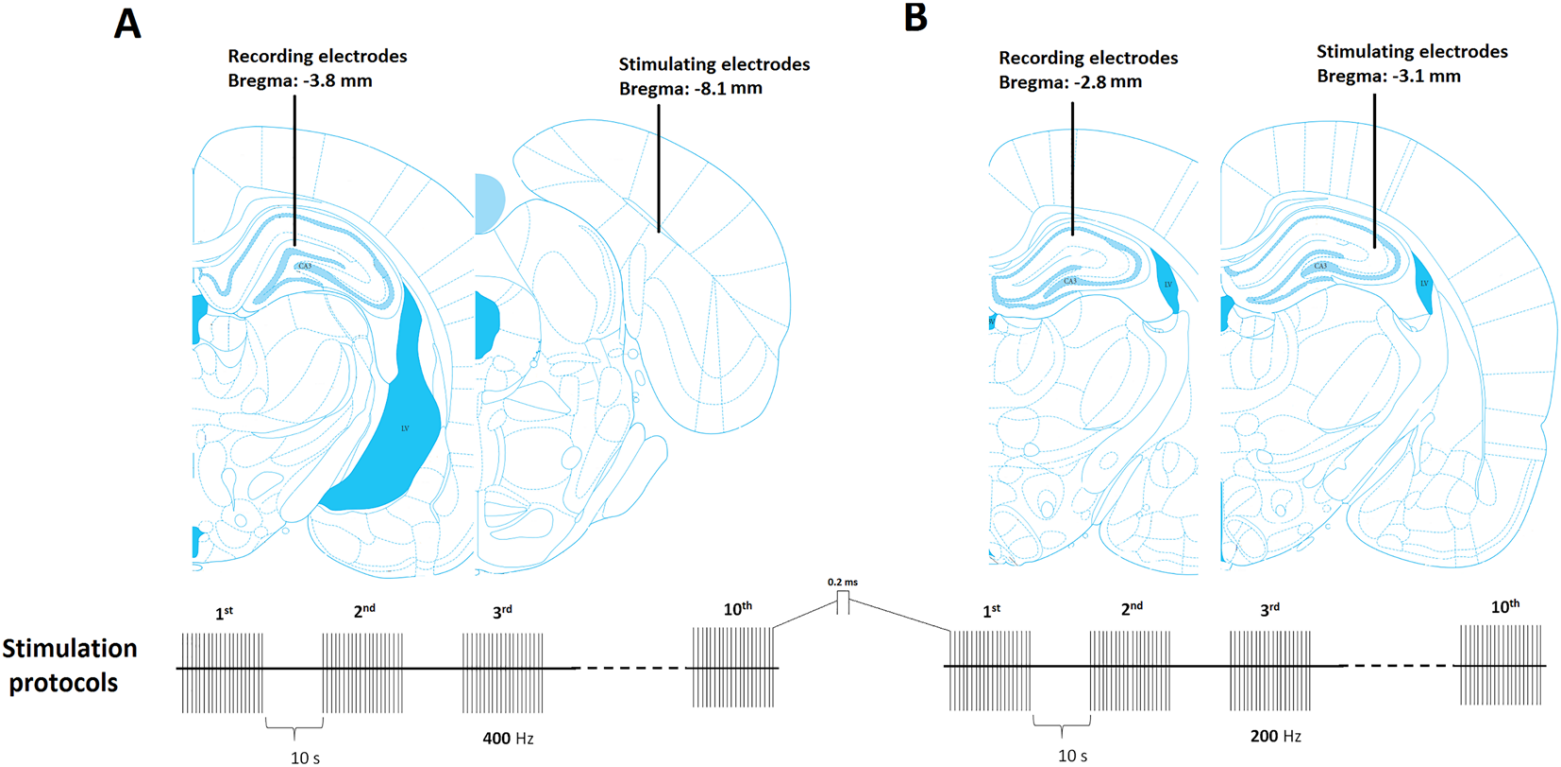
Schematic representations of stimulating and recording electrode positions for local field potential recording in DG (**A**) and CA1 (**B**) adapted from atlas of Paxinos and Watson. The stimulation protocol for each region is shown in the lower panel.

In vivo field potentials were recorded according the previously established method^30^. In order to record from DG, a bipolar stimulating electrode (A-M Systems, USA) was implanted at the perforant path (AP: 8.1 from bregma, ML: 4.3 from bregma, DV: 3.3 from dura), and a monopolar electrode was fixed at DG stratum granulosum (AP: 3.8 from bregma, ML: 2.4 from bregma, DV: 3–3.5 from dura) as recording electrode. In order to record from CA1, the bipolar stimulating electrode was fixed at Schaffer collaterals (AP: − 3.1 from bregma, ML: 3.1 from bregma, DV: 2.8–3.0 from dura), and a recording electrode was implanted at CA1 stratum radiatum (AP: − 2.8 from bregma, ML: 1.8 from bregma, DV: 2.6–2.8 from dura). Both electrodes were gently brought down. The pathway was stimulated until a classic synaptic signal was recorded. Thirty minutes were given to signal records be stabilized. A 0.2-ms monophasic square current was applied to record evoked field potentials. They were then amplified, filtered (between 1 Hz and 3 kHz) and digitized (10 kHz). The distance between the first crest and the trough point was considered as population spike (PS) amplitude. Field excitatory post-synaptic potential (fEPSP) slope was also determined from the first crest of EPSP before PS. PS and fEPSP are two separate events that were simultaneously recorded in one evoked local field potential. In order to find optimum stimulus, the PS amplitude upon various current intensities was obtained and the corresponding linear diagram (Input/Output curve) was drawn. The test stimulus producing half the maximum PS amplitude was defined as the optimum stimulus and selected for the main experiments. The optimum stimulus was applied every 5 min for 30 min to record baseline synaptic activity. For inducing long term potentiation (LTP), a HFS, consisting of 10 trains of 20 pulses at 400 Hz for DG^30,31^ and at 200 Hz for CA1^32^, with 80% of maximum intensity was delivered every 10 s, (Fig. 1). An increase of at least 25% in PS amplitude, compared to the baseline, was considered as the criteria of LTP induction. After applying HFS, the synaptic responses were recorded every 5 min for 90 min. The paired field potentials were elicited by paired-pulse stimuli with inter-pulse intervals (IPIs) between 10 to 200 ms. The PS amplitude of the second field potential was divided by that of the first one, and thus paired‐pulse ratio (PPR) was obtained.

### Experimental design

**Figure 2:**
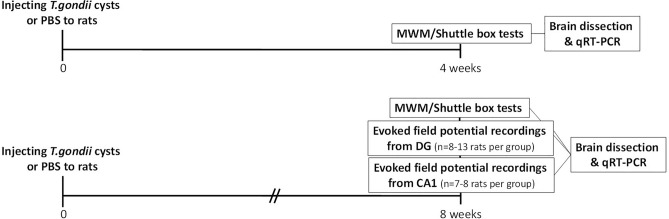
Timeline of the behavioral, electrophysiological and qRT-PCR experiments.

The experimental timeline is presented in Fig. 2. Experiments were performed in 2 steps. At first, spatial and avoidance learning/memory were assessed at 4 and 8 weeks after toxoplasma infection. Then evoked field potentials were measured in vivo in DG and CA1 of rats with an 8-week *T. gondii* infection. There was at least 6 rats in each experimental group. At the end of each step brain of rats was dissected out, and toxoplasma infection was assessed by RT-qPCR.

### Statistical analysis

All statistical analyses were performed using Graph Pad Prism 8 (GraphPad Software, CA, USA). Shapiro–Wilk test was used to examine normal distribution of data. Unpaired student’s t-test, and two-way ANOVA with Bonferroni multiple-comparison tests were used to analyze the data. Results were expressed as mean ± SEM (standard error of mean). The differences with *P* values less than 0.05 were considered statistically significant.

## Results

### Presence of the genomic DNA of *T. gondii* in brain of rats

RT-qPCR revealed presence of REP-529 genomic sequence in brain of all cyst-injected rats. The copy number of REP-529 was found from 10^2^ to 10^5^ per brain.

## *T. gondii* infection retarded spatial learning and memory performance in rats

**Figure 3:**
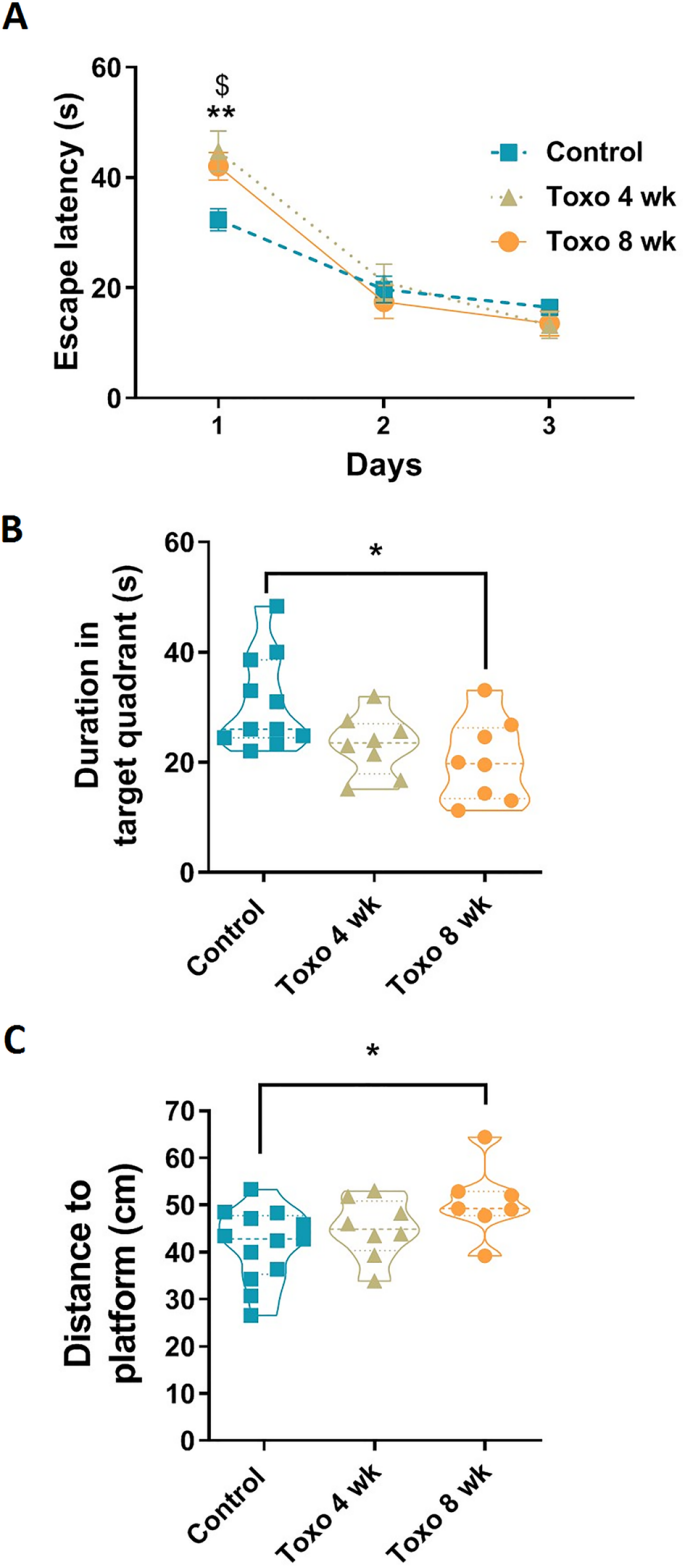
Spatial learning and memory performance in *T. gondii-*infected rats. (**A**) *T. gondii* increased the escape latency at 4 and 8 weeks after infection, but the infected rats found the platform on the 2nd and 3rd day with the same latency as the un-infected rats. (**B**,**C**) 24 h after the last training session probe test was done. Time spent in the target quadrant and distance to the platform were, respectively, decreased and increased in rats with an 8-week infection. No significant difference was found between un-infected rats and rats with a 4-week infection. *P < 0.05 and **P < 0.01 control vs. Toxo 8 wk, ^$^*P* < 0.05 control vs. Toxo 4 wk.

*T. gondii* reduced speed of spatial learning in rats four weeks after infection. As shown in Fig. 3A, rats with four and eight weeks infection, needed more time to escape and find the hidden platform than the control group on the first day of the task acquisition (*P* < 0.05 and *P* < 0.01, respectively). Yet, there was no significant difference between the groups on the day 2 and 3, indicating that the infected rats finally found the platform position in the tank. Testing memory function indicated that *T. gondii* decreased the time spent in the target quadrant and increased mean distance from the platform position in the probe test so that the changes reached the significant level at eight weeks after infection compared to the control group (*P* = 0.016 and *P* = 0.025, respectively, Fig. 3B,C).

**Figure 4:**
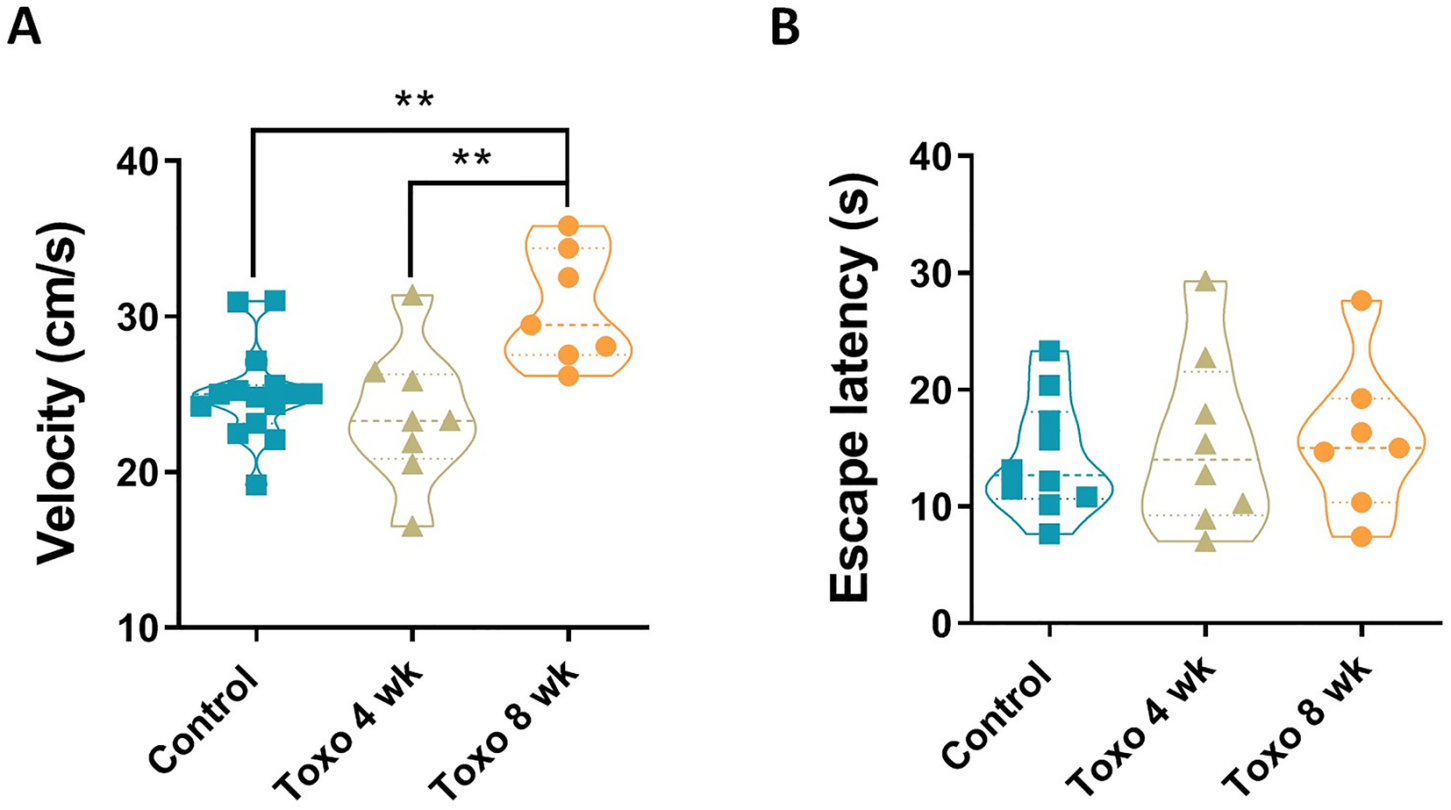
Effect of *T. gondii* infection on rats’ locomotion and vision performance. (**A**) Rats with an 8-week infection significantly swam with higher speed than rats with a 4-week infection, or no infection. No difference was found between control and Toxo 4 wk group. (**B**) No difference of escape latency to visible platform was found between groups. **P < 0.01.

The increased elapsed duration in the target quadrant or increased distance to platform position maybe because of the decreased motor activity in the infected rats. Therefore, visible test was conducted in which rats should find a visible platform during 60 s over 4 trials. The rats with an 8-week infection showed higher swimming speed than rats with no infection or a 4-week infection (*P* < 0.01). No difference was found between control and the 4-week infected groups (Fig. 4A). The parasite also did not affect vision ability, as all rats were on the visible platform in the same time range (Fig. 4B).

**Figure 5:**
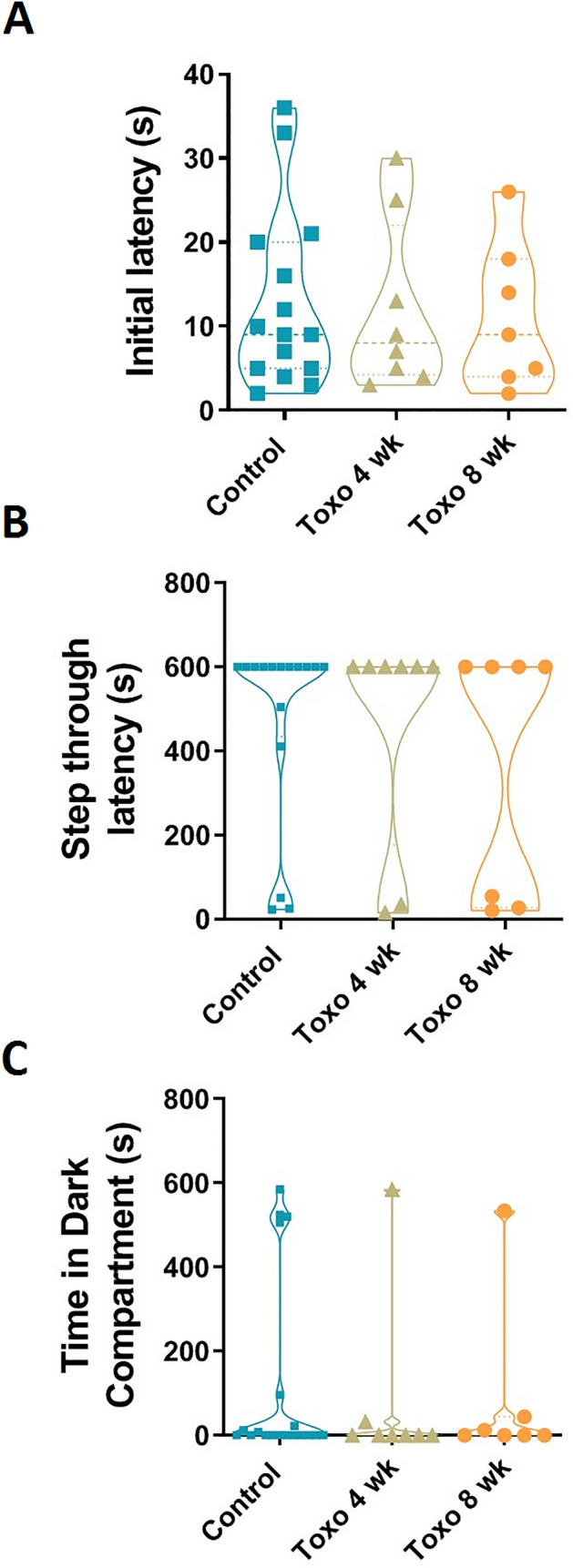
Passive avoidance task in *T. gondii-*infected rats. (**A**) All groups entered to the dark compartment with the same latency at the first exposure to the apparatus. (**B**) Step through latency to the dark compartment, where the rats received foot-shock 24 h earlier, did not differ among groups. (**C**) No difference was found among groups in total time spent in the dark compartment.

We did not find any significant effect of the parasite on the performance of inhibitory avoidance. Figure 5A shows that no difference was found between experimental groups in initial latency indicating the same motivation to enter the dark compartment in all groups. There was also no significant difference in step-through latency and time elapsed in the dark compartment among groups (Fig. 5B,C).

### The granule cell population spike is enhanced, following the induction of short- and long-term synaptic plasticity in rats with an 8-week *T. gondii* infection

**Figure 6:**
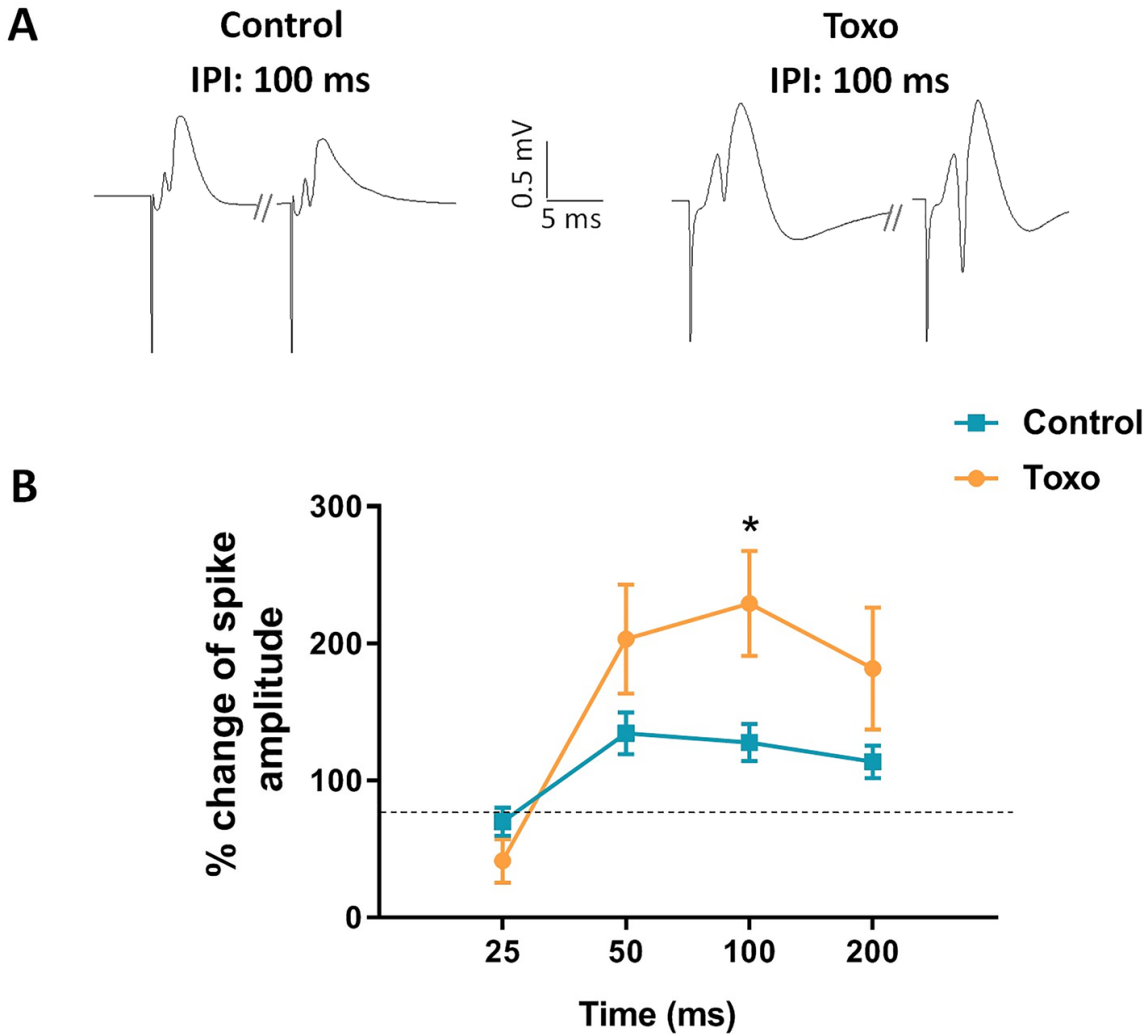
Network inhibition in *T. gondii*-infected rats. (**A**) Representative of post-synaptic response to double pulse stimulation at 100 ms inter-pulse interval (IPI) for un-infected and *T. gondii*-infected rats. (**B**) At 25 ms IPI, both un-infected and *T. gondii*-infected rats showed PPD. However, at greater IPIs, *T. gondii*-infected rats showed more facilitated response than un-infected rats and the difference reached significant level at 100 ms IPI. *P < 0.05 compared to control group.

According to Figs. 3 and 4, it was found that *T. gondii*-infected rats showed deficit in spatial learning and memory. Synaptic plasticity underlies the cellular mechanism of learning and memory. Therefore, STP and LTP in DG was investigated in experimental groups following stimulation of perforant pathway. We assessed STP in the DG by measuring paired-pulse depression/facilitation (PPD and PPF) at different IPIs. STP was examined before applying any HFS to the perforant pathway^33^. PPD mirrors the recruitment of GABAergic interneurons inhibiting granule cell by both feedback and feedforward mechanisms, whereas PPF or paired-pulse disinhibition (PPDI) is due to the inhibition of these interneurons. Figure 6A shows representative traces of double evoked field potentials at 100 ms IPI in control and *T. gondii*-infected rats. As shown in Fig. 6B, both un-infected and *T. gondii*-infected rats showed PPD at lower IPI (25 ms). However, at higher IPIs, *T. gondii-*infected rats showed more facilitated response compared to un-infected rats, and the difference reached significance level at 100 ms IPI (*P* < 0.05).

**Figure 7:**
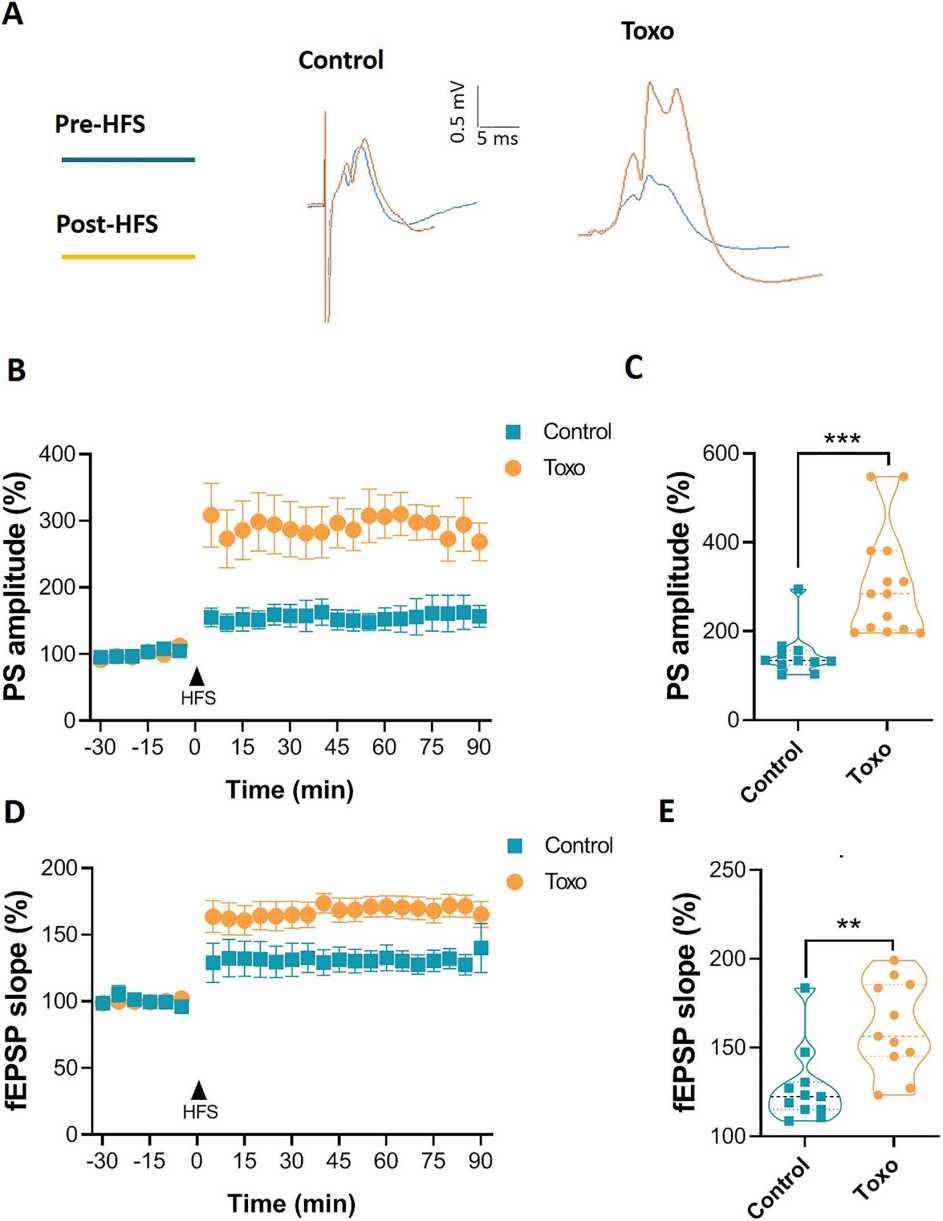
Enhanced LTP of PS and fEPSP slope at perforant pathway to granule cell synapses in *T. gondii*-infected rats. (**A**) Representative traces of evoked field potentials in the dentate gyrus recorded before and after HFS. The orange line shows the potentiated fEPSP at 30 min post-HFS period. (**B**) Time course of the PS-LTP in the experimental groups (n = 10–13). (**C**) Comparison of the mean post-HFS population spikes in control and *T. gondii*-infected rats during whole 90 min recording period. (**D**) Time course of the fEPSP slope-LTP in the experimental groups (n = 7 for each group). (**E**) Comparison of the mean post-HFS fEPSP slope in control and *T. gondii*-infected rats during whole 90 min recording. **P < 0.01, and ***P < 0.001.

LTP at perforant path to DG granule cells (PP-GC) synapses in *T. gondii*-infected and control rats was induced by HFS protocols. The 50% of max stimulation intensities were used to evoke a stable PS. Figure 7A shows representative post synaptic responses of DG to the perforant path stimulation before and after HFS. The pre-HFS PS amplitudes were smaller in *T. gondii-*infected rats compared to the control group (0.608 ± 0.059 mV vs. 2.046 ± 0.097 mV, unpaired t test, *P* < 0.0001); however, the post-HFS PS amplitudes were significantly increased in *T. gondii*-infected rats compared to the un-infected rats (Fig. 7B, two-way ANOVA with Bonferroni multiple-comparison tests, [F (1, 379) = 157.1, *P* < 0.0001). The responses remained potentiated during whole 90 min post-HFS period. The percentage change of the mean PS amplitude was significantly greater in *T. gondii* in contrast to control group (Fig. 7C, 14T*. gondii-*infected rats vs. 11 un-infected rats, 306.5 ± 32.32% vs. 148.2 ± 15.81%, respectively, unpaired t test, *P* < 0.0001). The fEPSP slope also increased after applying HFS to *T. gondii*-infected rats compared to the control group (Fig. 7D, two-way ANOVA with Bonferroni multiple-comparison tests, [F (1, 12) = 6.923, *P* = 0.0219]. A significant increase of the mean post-HFS fEPSP slope was found in *T. gondii*-infected group compared with control group (Fig. 7E, 161.8 ± 7.747 mV/s vs. 127.6 ± 6.467 mV/s, unpaired t test, *P* < 0.0029).

### EPSP-spike coupling is enhanced in *T. gondii*-infected rats

**Figure 8:**
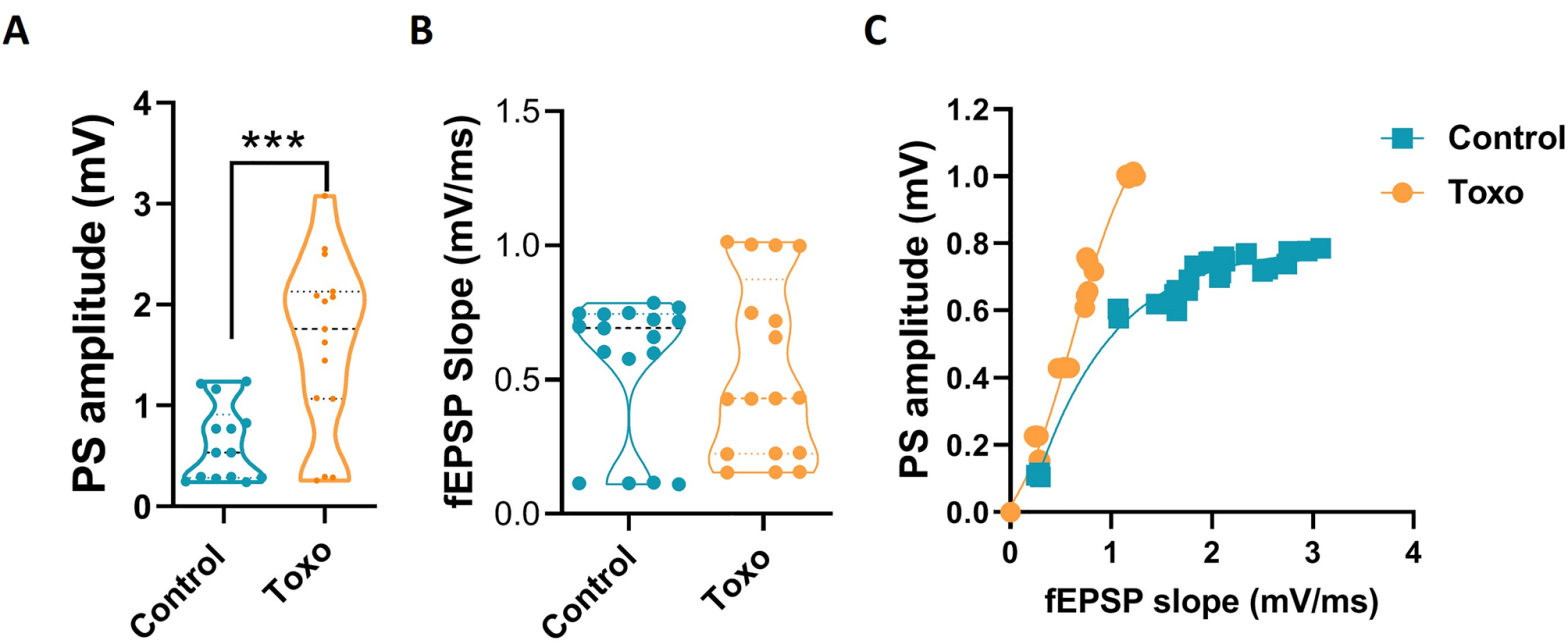
Spike-slope fEPSP relationship in *T. gondii*-infected rats. (**A**) The basal PS amplitude of fEPSP was significantly enhanced by *T. gondii* infection. (**B**) No difference was found between groups in the slope. (**C**) Spike-slope correlation fitted by Boltzmann function. Infection with *T. gondii* shifted the curve to the left. ***P < 0.001.

The synaptic strength (fEPSP slope) and the excitability of the DG granule cell population (PS) can be determined separately by the recording protocol used in this study; therefore, the superimposed PS on the fEPSP reflects the summed action potential firing activity of the granule cell population. Hence, the DG granule cell excitability was assessed in un-infected versus *T. gondii-*infected rats by determining the PS amplitudes using electrical stimulation with 50% maximum intensity. Comparing the results from both groups (Fig. 8A), there was significant difference in PS amplitude between control and infected groups (unpaired *t*-test, *P* < 0.0001), suggesting an enhanced capability of DG granule cells to fire action potentials in *T. gondii*-infected rats. However, no significant difference of EPSP slope was found between groups (Fig. 8B).

The relationship between synaptic input strength and capability of DG granule cells to fire action potential, was also assessed by plotting EPSP slopes versus PS amplitudes (Fig. 8C). The coupling between EPSP slopes versus PS amplitudes represents the DG granule cells intrinsic excitability as well as the local circuit excitation/inhibition balance. This analysis showed a leftward shift and a higher top parameter of the Boltzmann-fitted *T. gondii* curve (Fig. 8C, control vs. *T. gondii-*infected rats, 0.78 vs. 1.2) in contrast to the control group curve, indicating that the infected rats need a weaker synaptic input to fire similar spikes. In other words, they showed increased EPSP slopes-PS amplitudes coupling. On the other hand, the increased top parameter indicates the higher maximal PS amplitude in *T. gondii-*infected rats rather than control group. Therefore, it seems translation of synaptic input changes into firing responses was increased by *T. gondii.*

### *T. gondii*-potentiated synaptic plasticity in the hippocampal CA1

**Figure 9:**
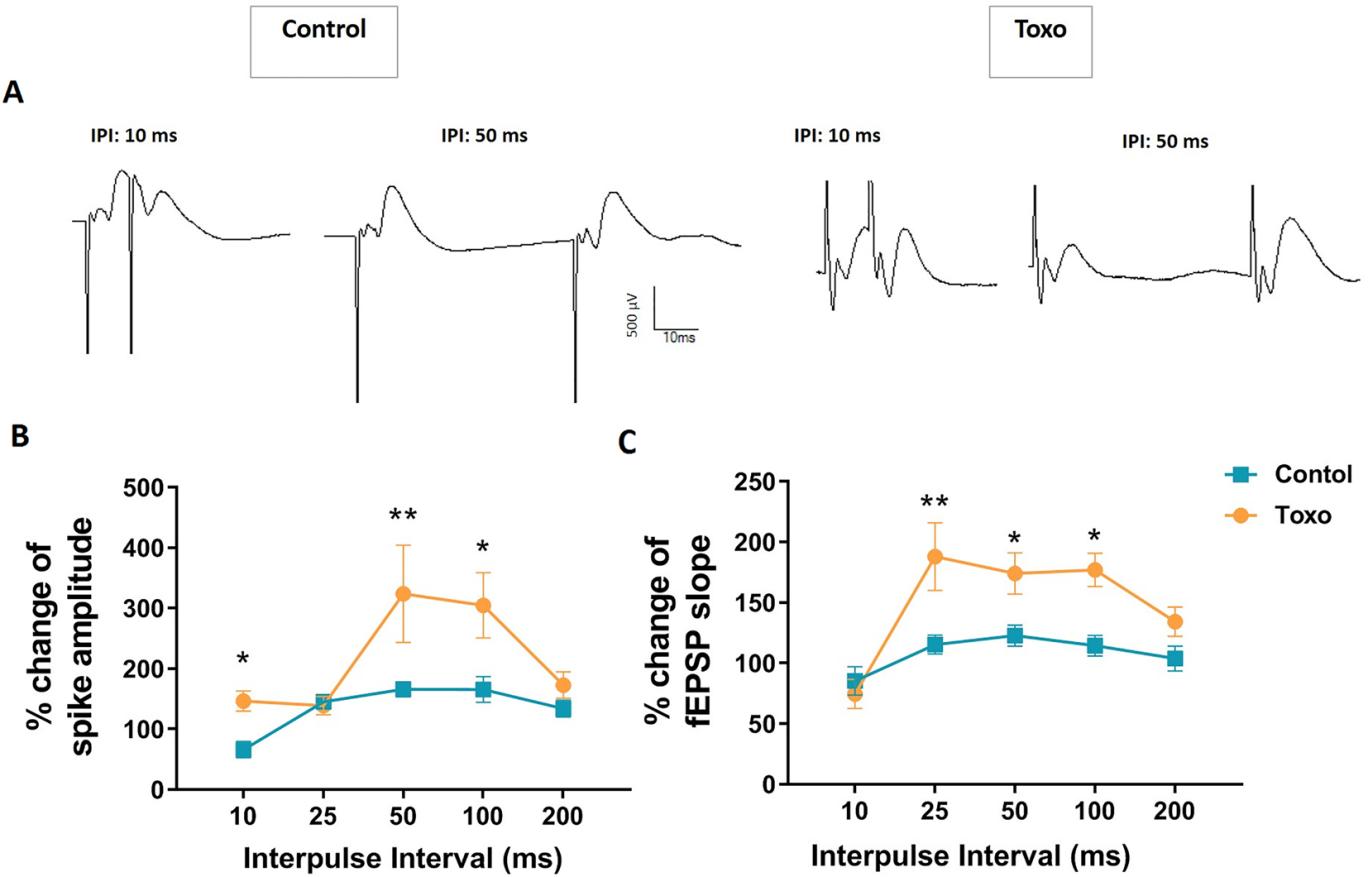
CA1 network inhibition in *T. gondii*-infected rats. (**A**) Representative of post-synaptic response to double pulse stimulation at 10 ms and 50 ms inter-pulse interval (IPI) for control and *T. gondii*-infected rats. (**B**) At 10 ms IPI, uninfected rats showed PPD of PS amplitude, but *T. gondii*-infected rats developed PPF. (**C**) Both un-infected and *T. gondii*-infected rats showed PPD of fEPSP slope. However, at greater IPIs, *T. gondii*-infected rats showed more facilitated response than un-infected rats and the difference reached significance level at 25–100 ms IPI. *P < 0.05, and **P < 0.01 compared to control group.

Despite disrupting behavioral performance, *T. gondii* enhanced synaptic plasticity in the DG. To confirm this result, we also assessed the synaptic plasticity of the CA1 region, as a main region involved in spatial memory. Short-term synaptic plasticity in the CA1 was determined by measuring PPD and PPF at different IPIs. Figure 9A shows representative of CA1 post-synaptic responses of un-infected and *T. gondii*-infected rats, to double pulse stimulation at 10 ms and 50 ms IPI. Unlike un-infected rats, *T. gondii*-infected rats showed PPF following double stimulation of Schaffer collaterals at lower IPI (10 ms) (Fig. 9B). At higher IPIs, both control and *T. gondii-*infected rats showed PPF but the PPF in *T. gondii-* infected rats was more robust than un-infected rats and the difference reached significance level at 50 and 100 ms IPI (*P* < 0.01 and *P* < 0.05, respectively). Figure 9C shows fEPSP slope in response to Schaffer collaterals double stimulation at different IPIs. Both un-infected and *T. gondii-*infected rats showed network inhibition at 10 ms IPI. At greater IPIs, *T. gondii-*infected rats had significantly higher PPF than control group.

**Figure 10:**
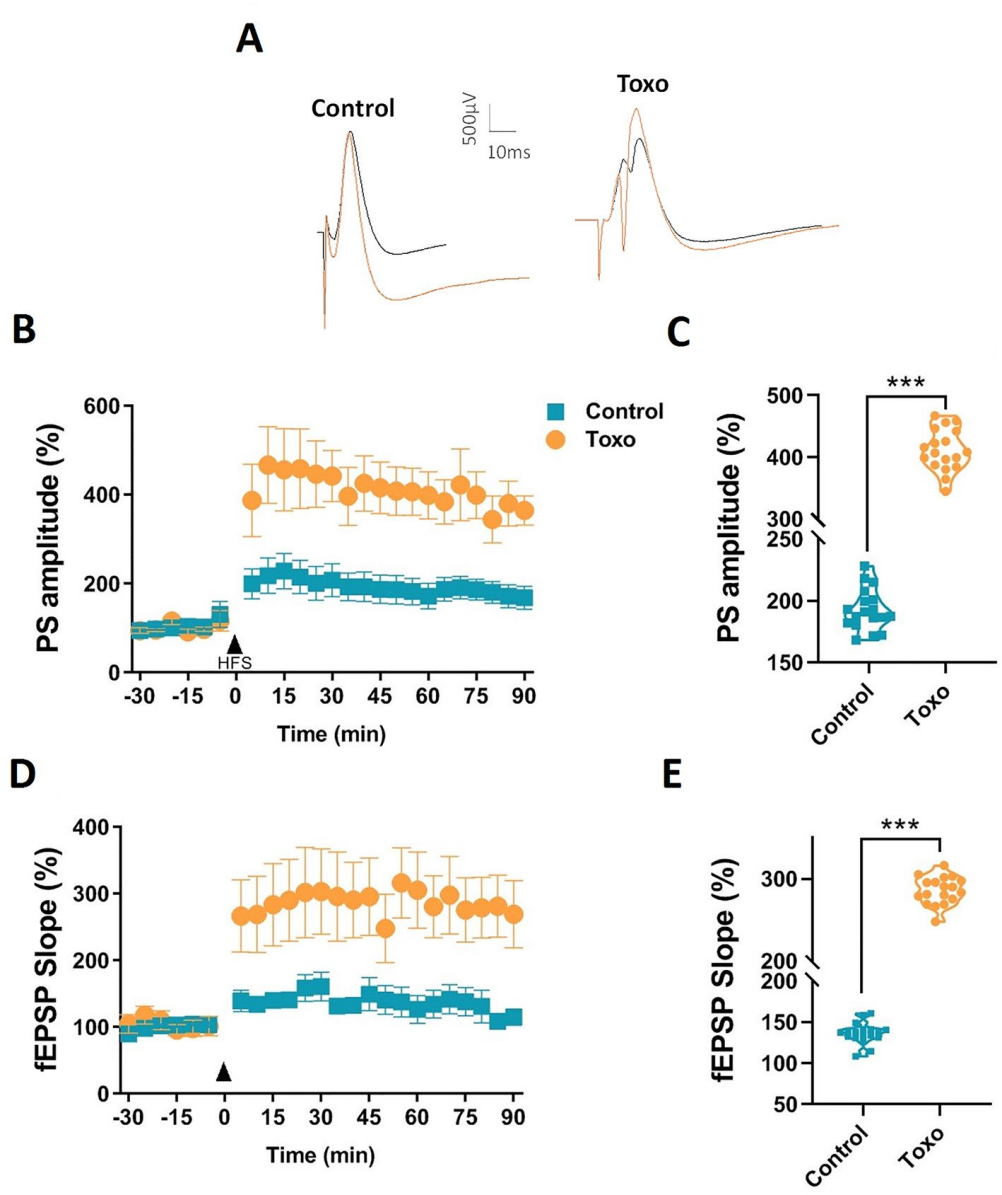
Enhanced LTP of PS and fEPSP slope at Schaffer collaterals to CA1 synapses in *T. gondii*-infected rats. (**A**) Representative traces of evoked field potentials in the CA1 recorded before and after HFS. The orange line shows the potentiated fEPSP at 30 min post-HFS period. (**B**) Time course of the PS-LTP in the experimental groups (n = 8 for each group). (**C**) Comparison of the mean post-HFS population spikes in un-infected and *T. gondii*-infected rats during 90 min recording period. (**D**) Time course of the fEPSP slope-LTP in the experimental groups. (**E**) Comparison of the mean post-HFS fEPSP slope in un-infected and *T. gondii*-infected rats during whole 90 min recording period (n = 7 for each group). Data are presented as mean ± SEM. ***P < 0.001.

Figure 10A shows representative post synaptic responses of CA1 to the Schaffer collaterals stimulation before and after HFS. Post-HFS PS amplitudes were significantly increased in *T. gondii-*infected rats compared to un-infected ones (Fig. 10B, two-way ANOVA with Bonferroni multiple-comparison tests, [F (1, 250) = 160.7, *P* < 0.0001). The responses remained potentiated during entire 90 min post-HFS period (Fig. 10C). EPSP slope also significantly increased in the *T. gondii*-infected rats in contrast to the un-infected rats (Fig. 10D, two-way ANOVA with Bonferroni multiple-comparison tests, [F (1, 266) = 84.53, *P* < 0.0001)]. The percentage change of post-HFS PS amplitude (Fig. 10C) and mean fEPSP slope (Fig. 10E) were significantly greater in *T. gondii*-infected rats than those of un-infected rats during whole 90 min post-HFS period.

### EPSP-spike coupling is enhanced in *T. gondii*-infected rats

**Figure 11:**
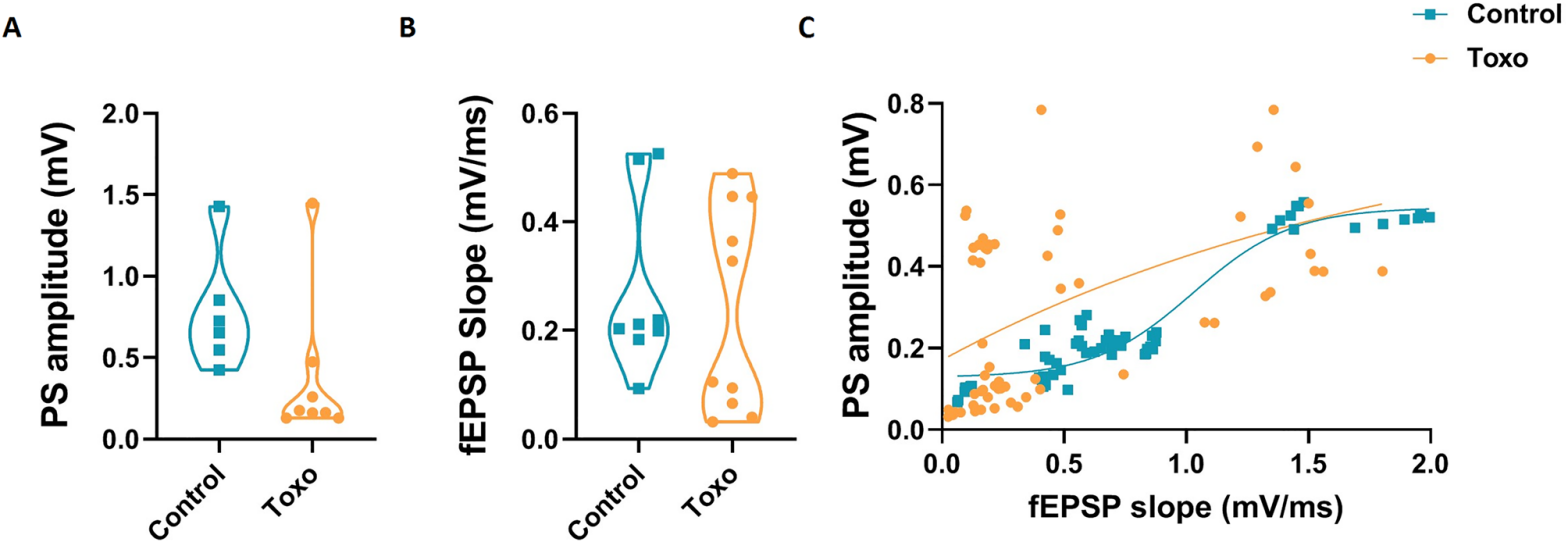
Spike-slope fEPSP relationship at CA1 of *T. gondii*-infected rats. Neither the basal PS amplitude (**A**) nor fEPSP slope (**B**) was changed by *T. gondii* infection. (**C**) Spike-slope correlation fitted by Boltzmann function. Infection with *T. gondii* shifted the curve to the left. Data are presented as mean ± SEM, (n = 6–10).

No Significant difference of basic PS amplitude or EPSP slope was found between control and infected groups (Fig. 11A,B unpaired *t*-test, *P* > 0.05). The relationship of synaptic input strength and capability of CA1 cells to fire action potentials was also determined by plotting PS amplitudes versus EPSP slopes (Fig. 11C). This analysis also showed a leftward shift and a higher top parameter of the Boltzmann-fitted *T. gondii* curve (Fig. 11C, control vs. *T. gondii*-infected rats, 0.54 vs. 0.8) in contrast to the control group curve indicating that *T. gondii*-infected rats require a weaker synaptic input to fire similar spikes. In other words, they showed increased EPSP slopes-PS amplitudes coupling. It seems translation of synaptic input into firing responses was also increased in the CA1 of *T. gondii-*infected rats.

## Discussion

In the present study, *T. gondii*-infected rats showed decelerated acquisition of the spatial task at both four and eight weeks after infection. They also developed deficit in spatial memory at eight weeks post-infection period. Since there was no difference in escape latency to locate visible platform between infected and uninfected control rats, the spatial learning and memory impairment cannot be attributed to defects of visual system. In the probe test, the infected rats showed a significant higher swimming speed at eight weeks post-infection period, indicating a higher motor activity compared to uninfected rats. Surprisingly, toxoplasma infection reinforced LTP in both DG and CA1 indicating the negative relationship between spatial memory and LTP in the infected rats.

There are several studies in line with our finding on destruction of learning and memory by toxoplasmosis. Mahmoudvand et al., reported that *T. gondii* aggravates spatial learning and memory deficits in mice^34^. Zhou et al., have showed an impairment of both reference and working spatial learning and memory in mice with latent *T. gondii* infection^35^. Also, Daniels et al., revealed that *T. gondii*-infected rats develop reference spatial recall while they show intact acquisition^36^. It is believed that *T. gondii* alters rodent behavior to transmit conveniently to feline definitive host mainly through reducing the undesired reaction to cat odors, which might subsequently increase the chance of predation^37^.

In this study, we did not find significant changes in the avoidance memory of the *T. gondii-*infected rats in the shuttle box test. Aversive response may just occur to a specific types of stimuli that facilitate the transmission of the parasite to its definitive host. In agreement to our finding, Bezerra et al., reported that passive avoidance memory is not affected in mice infected with two genetically modified *T. gondii* strains^38^. Moreover, it is found that early and intermediate, but not late, congenital infection with *T. gondii* lead to impairment of inhibitory memory performance in adult mice^39^. Passive avoidance is a fear-derived learning task which the anxious state can increase, impair, or not change acquisition of this task^40–43^. In current study, we did not specifically assess the anxiety-like behaviors in the infected rats. Regarding the impact of *T. gondii* on the level of anxiety, there are three types of reports including increase, decrease, and no change in anxiety-like behavior in the *T. gondii*-infected mice/rats^44–48^. Several factors such as load of parasite’s cyst, the period after infection in which the behavioral assessment was made, and the method of infecting the animal, have been implicated in these contradictory results. In order to further evaluate the relevance of anxiety state with acquisition of passive avoidance memory in animals with latent toxoplasmosis, the anxiety level of the infected rats needs to be determined.

The dorsal hippocampus is an essential brain region for acquiring and storing episodic memories, including spatial learning and memory. Synaptic plasticity in the neural circuits of the hippocampal formation is believed as a principal cellular mechanism underlying learning and memory formation. We observed that despite declined learning and memory ability, LTP is enhanced in the CA1 and DG synapses of *T. gondii-*infected rats. Although positive correlation between hippocampal LTP and memory formation is widely demonstrated^49^, there are numerous human^50,51^, and animal^52–57^ studies indicating enhancement of LTP, but impairment in the learning and memory performance. It is remarkable that most of the synaptic proteins involved in LTP augmentation/ learning deficit are also linked to the neuropsychiatric disorders with deficit in cognition, fear, social behaviors, and aggression^58–62^, which are also seen in toxoplasma-infected rodents. Therefore, it can be deduced that manipulation of synaptic proteins by *T. gondii* might underlie the learning deficit along with LTP augmentation in the infected rats. Similar to our finding, the coincidence of potentiated LTP and impaired spatial learning and memory has been reported in the autism candidate molecule, neurobeachin (Nbea) haploinsufficient (Nbea + /−) mice^63^. Meanwhile, it has been shown that constitutive activation of cAMP-responsive element-binding protein (CREB) in mice is associated with enhanced LTP and learning deficit in spatial task^64^. Interestingly, Nbea + / − mice developed enhanced LTP with increased phosphorylated CREB level in CA1 and DG, despite decreased spatial learning ability^63^. It is proposed that bi-directional and dynamic synaptic plasticity is critical to enhance storage capacity and optimize memory storage to decline memory errors. In line with this, Moser et al., have reported that overload of hippocampal LTP impairs spatial learning^65^. Postsynaptic density-95 (PSD-95) is an essential component involved in synaptic plasticity and LTP^66^. The impaired learning along with potentiated hippocampal LTP, have been previously reported in PSD-95-knockdown mice^53^. Interestingly, PSD-95 level is diminished in the hippocampus of the mice with chronic *T. gondii* infection^17^.

Markers of glutamatergic synapse function including the AMPA receptor subunits GluA1 and GluA2, the NDMA receptor subunits GluN1 and GluN2B, the glutamate transporter excitatory amino acid transporter 2 (EAAT2) and the postsynaptic scaffolding protein Shank3, as well as the GABA_A_ receptor α1 subunit as a marker of GABAergic function, which all are involved in the excitation/inhibition equilibrium in the CNS, are downregulated in the hippocampus of toxoplasma-infected mice^67,68^. All of these components enhance hippocampal LTP^69–72^. As EAAT2 is critical for clearance of extracellular glutamate from the synaptic cleft, and GABA_Aα1_ is a key component of inhibitory GABAergic signaling, their conjoint downregulation is likely to shift the delicate excitation-inhibition balance towards excitation, thereby promoting excitotoxicity^73^, and result in intensification of LTP in the *T. gondii*-infected animals.

In our study, analyzing the coupling of PS to EPSP slope shows left- and up-ward shift of the curve, indicating a dominant change of firing rate over EPSP of the post-synaptic neurons in the hippocampus of infected rats. This finding seems to be the electrophysiological explanation of decelerated learning and impaired memory performance of the infected rats. In agreement with this, Migaud et al., reported that PSD-95-mutant mice showed an inappropriate leftward and upward shift of the frequency-fEPSP slope function so that the mutant mice expressed LTP at diverse frequencies especially at low frequency that normally induced long-term depression (LTD) in wild type mice^53^. We did not examine stimulation the hippocampal pathways with low frequencies, but the same may have happened in our model. In our study, *T. gondii*-infected rats exhibited unidirectional synaptic enhancement, whereas synapses with bidirectional modification, in intact uninfected rats, are able to enhance storage capacity, decline errors, and optimally control the number and extent of strengthening synapses for maximal memory retention^74^. It has been reported that neurons in the hippocampus fire 4–12 Hz (theta range) during exploratory activation^75^. In this firing range, neurons in *T. gondii*-infected rats may show remarkable LTP, since they are near the border between LTD and LTP induction. The enhanced LTP has been shown to correlate with aberrant hippocampal place cell activity^76^. Potentiation of LTP may lead to an inappropriate change in synaptic weight of the neural networks during the acquisition (learning), network saturations and ultimately the declined capacity to acquire new tasks^64^. Therefore, the facilitated LTP reflects hyper-excitability of the principal hippocampal cells that may saturate the hippocampal neuronal networks and then manifest as learning/memory deficits.

The HFS patterns that we used in the present study (400 and 200 Hz for DG and CA1, respectively) were based on the previous studies of ours and others^77–79^, and can induce NMDA (N-methyl-D-aspartate)-dependent LTP that is normally mediated by Ca^2+^ influx through NMDA channel into post synaptic neurons in hippocampal CA1^80^ and DG^81^. Therefore, the LTP strengthening observed following infection with *T. gondii* in our study, can be induced by direct or indirect manipulation of postsynaptic NMDA receptors^82^. However, previous studies contradict this theory as it has been reported that *T. gondii* induces anti-NMDA receptor activity by immune cross reaction with the NMDA receptors^83^ and down-regulation of NMDA receptor subtypes in the brain^84^. Beside NMDA receptor, another components might be involved in LTP enhancement by *T. gondii* including activation of metabotropic glutamate receptors and/or voltage gated Ca^2+^ channels. Accordingly, HFS (200 Hz) of the Schaffer collaterals has been shown to add a component of voltage-dependent Ca^2+^ activation to the LTP in CA1 area^78^.

In addition to LTP, STP in both DG and CA1 synapses were also affected by *T. gondii* in the current study. We found that paired-pulse stimulation with 10 ms IPI to the Schaffer collaterals, which normally induces PPD, induced PPF in *T. gondii*-infected rats. At the greater IPIs, more robust PPFs were observed in *T. gondii*-infected group indicating an inhibitory network disruption in the hippocampal formation. It is worth noting that PPI observed in field potential recordings is normally translated to the hippocampal network inhibition^85^. It is mediated by feed-forward and feedback inhibition of hippocampal principal neurons by local GABAergic interneurons. Consistent with our finding, multiple evidence indicate that *T. gondii* modifies local GABAergic network in the brain. In this line, *T. gondii* infection alters the distribution of glutamic acid decarboxylase 67, the enzyme that catalyzes GABA synthesis in inhibitory synapses, so that a redistribution of presynaptic organization in inhibitory neurons or a loss of inhibitory nerve terminals occur^13^. Additionally, Carrillo et al., have reported that toxoplasma infection leads to a significant loss of perisomatic inhibitory synapses in neocortex and hippocampus^86^. They proposed a loss of inhibitory synapses following latent *T. gondii* infection, probably by ensheathment of GABAergic nerve terminals via microglia^86^. This may contribute to developing higher risk of seizures and schizophrenia in the infected individuals^86^.

In our study rats are infected by *T. gondii* at the age of 4 weeks. Then the hippocampal synaptic plasticity was assessed 8 weeks later at the age of 12 weeks. Given the alteration in NMDA receptor function and expression during development, and therefore different windows of NMDA receptor-dependent synaptic plasticity in the hippocampus^87–90^, it is likely that the different results might be obtained if hippocampal synaptic plasticity is assessed in the infected rats with ages less or more than 12 weeks. Moreover, there are evidence of non NMDA receptor-dependent LTP and LTD in the brain regions such as somatosensory cortex^91,92^. Therefore, studying effect of *T. gondii* on synaptic plasticity in different courses of development, and in different brain areas are required to achieve a broader perspective of the effect of *T. gondii* on the strength and efficacy of synaptic transmission.

Multiple evidence indicate that *T. gondii* is associated with behavioral changes through modulation of dopaminergic system^93–96^. It is well known that dopamine is one of the most important modulators of the hippocampal-dependent learning and synaptic plasticity^97^. Therefore, over-activity of dopaminergic system in latent toxoplasmosis might also be involved in the augmented induction of LTP at perforant pathway-DG, and Schaffer collatterals-CA1 synapses. Pharmacological manipulation of dopaminergic system is needed to prove this proposal.

In conclusion, in vivo electrophysiological findings of the present study further disclose the interaction of toxoplasma parasite with the host brain at synaptic level. The nature of changes in STP and LTP by *T. gondii* mimics almost a similar pattern seen in some mental diseases. These data will help to further analyze mental and cognitive alterations of the individuals with latent toxoplasmosis. In addition, our findings further confirm the deep impact of *T. gondii* on dynamic remodeling of the brain.

## Data Availability

All data are available from corresponding author upon reasonable request.
